# Is black carbon associated with cardiovascular and cancer mortality? Evidence from the Malmö Diet and Cancer Cohort

**DOI:** 10.1080/16549716.2026.2636879

**Published:** 2026-03-05

**Authors:** Cale Lawlor, Ebba Malmqvist, Daniel Oudin Åström, Tanya Andersson Nystedt, Jule Bamberger, Kajsa Pira, Rina So, Jiawei Zhang, Youn-Hee Lim, Zorana J. Andersson, Leo Stockfelt, Anna Oudin

**Affiliations:** a Division of Occupational and Environmental Medicine, Lund University, Lund, Sweden; b Section of Environmental Health, Department of Public Health, University of Copenhagen, Copenhagen, Denmark; c Occupational and Environmental Medicine, School of Public Health and Community Medicine, Institute of Medicine, Sahlgrenska Academy, University of Gothenburg, Gothenburg, Sweden; d Department of Occupational and Environmental Medicine, Sahlgrenska University Hospital, Gothenburg, Sweden; e Department of Epidemiology and Global Health, Umeå University, Umeå, Sweden

**Keywords:** Black carbon, air pollution, cardiovascular, cancer, mortality

## Abstract

**Background:**

Black carbon (BC) is an air pollutant of growing concern due to its adverse impacts on health and climate. Growing evidence suggests that BC could have a number of negative impacts on morbidity and mortality, but more evidence is needed.

**Objective:**

The objectives of this study are to quantify any associations between BC exposure and cause-specific (cardiovascular and cancer) mortality outcomes.

**Methods:**

Using the Malmö Diet and Cancer Cohort linked with a high-resolution dispersion model, we examined the association between long-term exposure to locally emitted BC, nitrogen oxides (NO_x_) and fine particulate matter (PM_2.5_) with cardiovascular and cancer mortality.

**Results:**

In fully adjusted models, BC exposure was consistently associated with cardiovascular mortality (HR 1.15 [1.06–1.26] per IQR increase), an association that was stronger and more robust than for PM2.5 or NO_x_. This association was present across all models, all time periods and both sets of exposure intervals. This association was stronger than with the other pollutants. Less clear association was found between any pollutant and cancer mortality.

**Conclusion:**

This study shows associations between BC exposure and especially cardiovascular mortality, consistent with international evidence showing similar impacts. For cancer mortality, there were tendencies of an association with BC but less clear than for cardiovascular mortality. These findings suggest a unique role of BC in air pollution-related cardiovascular mortality and support the need for action on mitigation of air pollution in general and BC in particular.

## Paper context

- **Main findings:** Black carbon was associated with cardiovascular mortality, but not cancer mortality, in this cohort in a low pollution environment.
- **Added knowledge:** Black carbon was more strongly associated with cardiovascular mortality than other pollutants.
- **Global health impact on policy and action:** These findings strengthen the call for regulation of black carbon pollution for both health and environmental protection.

## Background

Black carbon (BC) is an emerging air pollutant of global concern, with a growing evidence-base suggesting an association between BC exposure and adverse health outcomes [1]. BC is a component of particulate matter, including fine particulate matter with an aerodynamic size of ≤2.5 μm (PM_2.5_) [2]. In addition to its health impacts, BC contributes to climate change through deposition which enhances solar heat absorption, particularly on snow and glaciers, and may also influence cloud formation. The climate effect will, however, depend, as it is always emitted with organic carbon, which has a cooling effect [3]. BC is emitted as the result of incomplete combustion of fossil and biomass fuels [4]. In Europe, the largest source is road traffic [5,6], with residential heating, commercial and institutional sources, and waste, being other significant contributors [6]. Biomass burning, wildfires, cooking, industrial processes and other forms of transport are also relevant [7–9]. Nitrogen oxides (NO_x_), another significant air pollutant, are often co-emitted from these sources. NO_x_ is an indicator of traffic emissions, and traffic is typically the major source of NO_x_ in urban areas [10].

BC is of increasing interest in epidemiological public health research due to accumulating evidence of its adverse effects on morbidity and mortality. Suggested biological pathways for BC-related health impact revolve around immune reactions to inhaled particles [11,12]. Once BC is inhaled, it has been shown to be able to translocate along anatomical structures, disseminate through circulation, cross vascular structures and deposit into tissues [4,13–16]. Like other forms of air pollution, the immune reaction to BC can set off a cascade of biochemical processes, which result in disease outcomes, including cardiovascular disease and cancer development and mortality [17].

A 2023 meta-analysis of the BC impact found a pooled, adjusted HR (RR) for long-term exposure to total mortality of 1.223 [95% confidence interval (95% CI): 1.023−1.462] for an increase of 10 μm/m^3^ [1]. Associations have been suggested for several cause-specific mortality outcomes in other studies, but findings have been varied [1]. Multiple studies have found positive suggestive associations between BC and mortality from cardiovascular diseases [18–23], ischaemic heart disease [18], myocardial infarction [24], respiratory diseases [18,21,22], lung cancer [18,21], chronic obstructive pulmonary disease (COPD) [18], chronic kidney disease [25], cardiometabolic diseases [18,23], type 2 diabetes [18], gastrointestinal malignancy [26], diseases of the nervous system [27] and cerebrovascular diseases [18,27]. Multiple studies have also found null-associations, however, including for mortality related to cardiovascular disease [28], ischaemic heart disease [23,27,29], respiratory disease [23,27], lung cancer [19,23], COPD [27], type 2 diabetes [18,27] and cerebrovascular disease [18,23]. Disentangling BC effects from the effects of co-emitted pollutants for total mortality, such as PM_2.5_, has been cited as a methodological issue in the research [1].

A recent meta-analysis highlighted the need for additional studies that evaluate BC as a separate pollutant in cause-specific mortality, specifically for cardiovascular disease and ischaemic heart disease, as well as cerebrovascular disease, and not just as a component of PM_2.5_ [1]. In line with this recommendation, the aim of this study is to assess the association between BC, NO_x_ and PM_2.5_ and cause-specific mortality in a well-established cohort using modelled air pollution with high spatial resolution and to examine the independent effects of BC on cause-specific mortality.

## Methods

### Description of cohort

The Malmö Diet and Cancer Cohort (MDC) is a large cohort of 30,446 participants residing in Malmö, Sweden, and born between 1923 and 1950. Between 1991 and 1996 all citizens of Malmö aged 45–73 years were invited to participate in the prospective cohort. A detailed questionnaire was used to collect information, such as demographic and socioeconomic variables, occupations, lifestyle choices (alcohol, diet, smoking, nutritional profile and physical activity) and medical history. A clinical examination was performed to collect data on blood pressure, weight and height and blood sampling [30]. Results from this cohort have been used for previous studies and publications, including on diet and activity, occupation and education, type 2 diabetes, cancer incidence, coronary events and carotid atherosclerosis [31–37]. We have permission to access this database and perform analyses.

### Exposure assessment

We modelled concentrations of BC, NO_x_ and PM_2.5_ for Malmö, covering an area of 18 km × 18 km. We focused on locally emitted pollutants to better isolate the within-city exposure gradients that are most relevant for informing local policy and urban planning, as long-range transport contributes to a more uniform regional background and as we have a highly detailed local exposure assessment based on the emission inventory. The modelling was based on a local emission database and a Gaussian plume dispersion model implemented in the ENVIMAN software; a local adaptation of the American Meteorological Society/Environmental Protection Agency Regulatory Model (AERMOD) [38]. The levels below refer to locally emitted pollutants based on local emissions data. Emissions from nearby areas in Denmark are included in the model, but long-range transported pollutants were not added.

Separate emission inventories were compiled for the years 1992, 2000 and 2011 using data provided by the municipality. For each pollutant, concentrations were estimated as the sum of local emissions from traffic exhaust, non-exhaust traffic emissions (mechanically generated particles from road, tire and brake wear, including resuspension), residential heating, shipping, industry, households and long-range transported emissions. A detailed description of the extensive emission data has been provided previously by Hasslöf et al. (2020) and Xu et al. (2022) [30,39] and is detailed down to each road segment on vehicle types, road density and speed. The emission factors for BC are based on well-established emission factors from, for example, the project TRANSPHORM (http://www.transphorm.eu/) for road traffic.

The annual mean modelled concentrations were stored as spatial grids with a resolution of 50 × 50 m. During model development, validation against measurements had been done for PM_2.5_ and NO_x_ with good agreements in the study area and correlations (*R*^2^) 0.86 for PM_2.5_ [40,41]. Similar validation could not be done for BC as historical measurement data was lacking.

To estimate concentrations for years between 1992, 2000 and 2011, interpolation was applied. These values were further adjusted for year-to-year meteorological variations based on data from a local weather station. In a sensitivity analysis, the concentrations 2012 to 2016 were extrapolated using the last observation carried forward.

We retrieved participants’ residential addresses from 1991 to 2016, geocoded by Statistics Sweden. For each year during this period, we modelled mean concentrations of PM_2.5_, NO_x_ and BC, which were assigned to each participant’s home address.

### Outcome variables

We defined mortality on the basis of the underlying cause of death according to the International Classification of Diseases, Tenth Revision (ICD-10: A00–R99). Cause of death was obtained from the first cause of death on a documented death certificate. All causes of cardiovascular and cancer mortalities were included as the outcome variables, capturing all ICD-10 codes for cardiovascular (ICD-10, I00-I) and cancer-related (ICD-10, C00-99) main causes of death, respectively.

The mortality outcomes – cardiovascular and cancer mortality – were selected based on the availability of a sufficient number of cases to ensure adequate statistical power for analysis.

### Statistical analyses

To account for the competing risk scenarios, we used a cause-specific Cox proportional hazard regression model to examine the association between air pollution exposure and cause-specific mortality, with time-varying exposures, expressed as hazard ratios (HRs) with 95% CIs. Individuals were followed from cohort baseline until death, emigration or end of follow-up, whichever came first. Timescale and exposure were counted and averaged by the calendar year, and follow-up time for participants was counted from recruitment date.

To account for potential confounders, we included birth year as a continuous variable and sex, as fundamental covariates. Socioeconomic factors were adjusted for by incorporating education level and relationship status (as a proxy for cohabitation). Relationship status was categorised as single/widowed and never married or married/cohabiting. Alcohol consumption was assessed based on self-reported intake, measured in grams per day. Physical activity levels were categorised as low, medium or high, and the waist-to-hip ratio was also considered as a covariate. Vegetable intake ranking was based on an intra-cohort distribution [42]. BMI was measured using the standard formula from physical examination. Other categorical covariates were modelled as follows:

- Occupational status was included as a categorical variable with five levels: ‘housewife’ (as labelled in the original survey, meaning domestic/house work), employed, retired, student and unemployed.
- Education level was as low: <9 years, medium: 9–12 years and high: >12 years
- Smoking status was modelled as: current smoker (occasional and regular), former smoker and never smoker.
- Year of enrolment and year of study as categorical covariates.
- All other covariates (BMI, physical activity score, alcohol consumption, pack-years of smoking, waist–hip ratio, vegetable consumption and relationship status) were included as continuous linear terms unless otherwise specified.

All the covariates were recorded at baseline (1991–1996). The exposure variables (BC, NO_x_ and PM_2.5_) were entered as continuous variables.

We adjusted all models for potential confounders. We fitted three sequential models to examine the robustness of the associations:

- Model 1: Adjusted for birth year and sex.
- Model 2: Additionally adjusted for employment status, education, physical activity score, smoking status, BMI, marital status, waist–hip ratio, alcohol consumption, year of enrolment, year of study and country of birth (Sweden/other countries).
- Model 3: Further adjusted for smoking pack-years and vegetable consumption.

Model 1 served as a crude model, adjusting for only birth year at enrolment and sex. Model 2 included additional adjustments for lifestyle factors, education level and cohabitation status. Model 3 further expanded on the covariates from models 1 and 2 by incorporating smoking and dietary habits. HRs and 95% CIs were obtained for each exposure.

All analyses were conducted separately for cardiovascular mortality and cancer mortality with a cause-specific Cox regression model. We assessed associations in single-pollutant models and did not include any estimates from two-pollutant models in the results due to high correlation coefficients (see Supplementary Material, Figure 1) and evidence of collinearity in these models. Two sets of exposure data were used: (1) limited to air pollution data up to 2011 and (2) extended to 2016 via imputation, where 2011 levels were extrapolated forward.

To capture the potential lagged effects of exposure on mortality, we calculated exposures using three time windows: the year of death (lag0), 1–5 years prior to death (lag1–5) and 6–10 years prior to death (lag6–10). Participants who had missing exposure data in their lag calculation were excluded from the analysis. Missing data among covariates are furthermore seen in Supplementary Material, Table 5 and Figure 2. For a sensitivity analysis, mortality follow-up was limited to 2011, aligning with the period for which air pollution exposure data were available

We calculated E-values to assess the robustness of the findings to potential unmeasured confounding. The E-value quantifies the minimum strength of association that an unmeasured confounder would need to have with both the exposure and the outcome, conditional on the measured covariates, to fully explain away the observed association. E-values were computed for the point estimates and the confidence interval limits using the approach described by VanderWeele and Ding [43]. For example, an E-value of 1.57 indicates that an unmeasured confounder would need to be associated with both the exposure and the outcome by a risk ratio of at least 1.57 each, above and beyond the measured covariates, to fully explain away the observed association. Unmeasured confounders with weaker associations are therefore unlikely to account for the observed effect. All analyses were done using R version 4.2.

The study was approved by the Regional Ethics Committee at the University of Lund (dnr 2016/4).

## Results

A total of 30,440 participants were included in the analysis. We conducted a complete case analysis, with varying complete cases in each model, depending on the number of missing values for each included variable. The numbers for each analysis are stated in the tables. During the span of the survey, 2135 cardiovascular deaths and 2614 cancer deaths were recorded. Levels of exposure across time on average were 0.41 µg/m^3^ for BC, 19.20 µg/m^3^ for NO_x_ and 1.70 µg/m^3^ for PM_2.5_. These levels refer to locally emitted pollutants, to be able to inform local policy action. Previous studies have shown that about 7% of PM_2.5_ is from local sources and 10% of NO_x_ is from long-range transported [44,45]. Descriptive statistics of the cohort can be found in Table 1. As expected, smoking and alcohol consumption was higher for those who died of cardiovascular or cancer mortality by the end of follow-up.

**Table 1.** Descriptive statistics of the cohort including continuous and categorical variables.

Variables		All cohort	Cardiovascular mortality		Cancer mortality	
			No	Yes	No	Yes
		*n* = 30440	*n* = 28305	*n* = 2135	*n* = 27826	*n* = 2614
Continuous variables – mean ± standard deviation						
Physical activity score		8086 ± 6647	8098 ± 6644	7921 ± 6700	8072 ± 6570	8238 ± 7424
BMI		25.8 ± 4.0	25.7 ± 4.0	26.8 ± 4.4	25.8 ± 4.0	26.0 ± 4.1
Waist/hip ratio		0.86 ± 0.15	0.85 ± 0.15	0.9 ± 0.09	0.85 ± 0.13	0.88 ± 0.25
Alcohol consumption (g/day)		10.7 ± 12.7	10.7 ± 12.5	10.3 ± 15	10.7 ± 12.5	11.2 ± 14.5
Smoking pack years		6.9 ± 16.2	6.7 ± 15.8	10.4 ± 21	6.5 ± 15.1	12.4 ± 24.6
Vegetable consumption		181 ± 101	183 ± 101	161 ± 92	183 ± 101	167 ± 93
Categorical variables – number (%)						
Sex	Male	12117 (39.8)	10859 (38.4)	1258 (58.9)	10805 (38.8)	1312 (50.2)
	Female	18323 (60.2)	17446 (61.6)	877 (41.1)	17021 (61.2)	1302 (49.8)
Education level	Low	19393 (68.1)	17897 (67.3)	1496 (78.1)	17564 (67.4)	1829 (74.7)
	Medium	5028 (17.6)	4775 (18.0)	253 (13.2)	4659 (17.9)	369 (15.1)
	High	4073 (14.3)	3906 (14.7)	167 (8.7)	3821 (14.7)	252 (10.3)
Cohabiting w. partner	No	9984 (35.0)	9214 (34.6)	770 (40.0)	9081 (34.8)	903 (36.9)
	Yes	18566 (65.0)	17412 (65.4)	1154 (60.0)	17019 (65.2)	1547 (63.1)
Current employment	Housewife^a^	501 (1.8)	477 (1.8)	24 (1.3)	467 (1.8)	34 (1.4)
	Employed	16693 (59.7)	16170 (61.9)	523 (28.3)	15626 (61.1)	1067 (44.5)
	Retired	9234 (33.0)	7992 (30.6)	1242 (67.2)	8050 (31.5)	1184 (49.4)
	Student	100 (0.4)	99 (0.4)	1 (0.1)	94 (0.4)	6 (0.3)
	Unemployed	1427 (5.1)	1370 (5.2)	57 (3.1)	1322 (5.2)	105 (4.4)
Smoking	Current	8087 (28.3)	7422 (27.9)	665 (34.6)	7142 (27.4)	945 (38.6)
	Former	9655 (33.8)	8952 (33.6)	703 (36.6)	8836 (33.8)	819 (33.4)
	Non	10817 (37.9)	10264 (38.5)	553 (28.8)	10130 (38.8)	687 (28.0)
Birth country	Sweden	28227 (92.7)	26231 (92.7)	1996 (93.5)	25769 (92.6)	2458 (94)
	Other	2213 (7.3)	2074 (7.3)	139 (6.5)	2057 (7.4)	156 (6)

Percentages are listed column-wise for the specified categorical variable per outcome. ‘*n*’ refers to the number of complete cases for the specified outcome. ^a^The word ‘housewife’ has been kept as it was asked in the original survey, though it is outdated terminology now. BMI – body mass index, g – grams.

In all models, across both sets of time periods and both sets of exposure intervals, BC exposure was associated with cardiovascular mortality in the lag0 analysis. Focusing on IQR as the exposure interval, which for BC in this data was 0.12 µg/m^3^, BC was associated with cardiovascular mortality in model 1 with an HR and 95% CI of 1.25 [1.17–1.35], 1.16 [1.06–1.27] in model 2 and of 1.15 [1.06–1.26] in model 3, for the years to 2011. Results for the IQR using data up to 2011 are available in Table 2. In models using data up to 2011 and not extrapolated data, BC exposure at 1 µg/m^3^ was associated with an HR for cardiovascular mortality of 4.31 with a 95% CI [2.72–6.85]. After adjustment in model 2, the HR was 2.59 with a 95% CI [1.45–4.61], and in model 3, 2.52 with a 95% CI [1.41–4.51]. Results using the 1 µg/m^3^ interval for years 2011 and 2016 are available in Supplementary Material, Tables 1 and 2, respectively. Results for the IQR interval to 2016 are available in Supplementary Material, Table 3.

**Table 2.** Associations between BC, NO_x_ and PM_2.5_ exposure lag0 and cardiovascular and cancer mortality, expressed as hazard ratios (HRs) with 95% confidence intervals, per IQR increase, based on non-extrapolated data up to 2011, with estimates being rounded to two decimal places.

	Model 1 *n* = 2103	Model 2 *n* = 1472	Model 3 *n* = 1452
Cardiovascular mortality			
BC	1.25 [1.17–1.35]	1.16 [1.06–1.27]	1.15 [1.06–1.26]
NO_x_	1.09 [1.02–1.17]	1.09 [0.99–1.19]	1.08 [0.99–1.19]
PM_2.5_	1.11 [1.03–1.20]	1.08 [0.98–1.20]	1.08 [0.97–1.19]
	Model 1 *n* = 2586	Model 2 *n* = 2057	Model 3 *n* = 2027
Cancer mortality			
BC	1.12 [1.05–1.20]	1.06 [0.99–1.14]	1.06 [0.98–1.14]
NO_x_	1.01 [0.95–1.08]	1.03 [0.95–1.10]	1.02 [0.94–1.10]
PM_2.5_	1.00 [0.93–1.08]	1.03 [0.95–1.12]	1.03 [0.94–1.19]

‘*n*’ refers to the number of complete cases for the specified outcome. BC – black carbon. NO_x_ – nitrogen oxides. PM_2.5_ – fine particulate matter. Results were presented as IQR increase for each pollutant, which is 0.12 µg/m^3^ for BC, 6.05 µg/m^3^ for NO_x_ and 0.56 µg/m^3^ for PM_2.5_. Model 1: year of birth and sex; Model 2: additional adjustment for employment status, education, physical activity score, smoking status, BMI, marital status, waist–hip ratio, alcohol consumption, year of enrolment, study year and country of birth; Model 3: further adjustment for smoking pack-years and vegetable consumption.

**Table 3.** Associations between BC, NO_x_ and PM_2.5_ exposure at lag1–5 and lag6–10 and cardiovascular and cancer mortality, expressed as hazard ratios (HRs) and 95% confidence intervals, per 1 µg/m^3^ increase, based on data up to 2011 in model 3, with estimates rounded to two decimal places.

	Lag0 *n* = 1452	Lag1–5 *n* = 1450	Lag6–10 *n* = 1285
Cardiovascular mortality			
BC	2.52 [1.41–4.51]	2.93 [1.63–5.27]	2.35 [1.28–4.32]
NO_x_	1.01 [0.99–1.02]	1.01 [1.00–1.02]	1.01 [1.00–1.01]
PM_2.5_	1.12 [0.96 - 1.30]	1.15 [0.98–1.35]	1.09 [0.95–1.28]
	Lag0 *n* = 2027	Lag1–5 *n* = 2025	Lag6–10 *n* = 1750
Cancer mortality			
BC	1.44 [0.89–2.32]	1.52 [0.94–2.46]	1.35 [0.81–2.26]
NO_x_	1.00 [0.99–1.01]	1.00 [0.990–1.01]	1.00 [0.99–1.01]
PM_2.5_	1.04 [0.91–1.18]	1.03 [0.91–1.17]	1.02 [0.90–1.15]

‘n’ refers to the number of complete cases for the specified outcome. BC – black carbon. NO_x_ – nitrogen oxides. PM_2.5_ – fine particulate matter. Model 3: year of birth and sex, employment status, education, physical activity score, smoking status, BMI, marital status, waist–hip ratio, alcohol consumption, country of birth, year of enrolment, study year, smoking pack-years and vegetable consumption.

Cancer mortality showed weaker associations with BC, with clear associations only found in model 1 in both sets of time periods, in both sets of exposure intervals in the lag0 analysis; the association was attenuated in models 2 and 3. For BC, the HRs and 95% CIs in models 1–3 were 1.12 [1.05–1.20], 1.06 [0.99–1.14] and 1.06 [0.98–1.14], respectively, per IQR increase, as shown in Table 2.

Associations between cardiovascular and cancer mortality, and NO_x_ and PM_2.5_, were mixed, but generally less consistent than those observed for BC. Clear associations with NO_x_ and PM_2.5_ were only seen in model 1, while significance disappeared in models 2 and 3. This pattern held across both time periods and for different exposure intervals. However, results for NO_x_ exposure and cardiovascular mortality were quite stable across the three models; HRs and 95% CIs were 1.01 [0.95–1.08], 1.03 [0.95–1.10] and 1.02 [0.94–1.10], respectively. Results for these data are presented in Table 2. As additional analyses, we imputed missing air pollution data up to 2016 using the last observation carried forward to include more cases; results changed only marginally (see Supplementary Material, Table 3). Data using 1 µg/m^3^ interval for BC, NO_x_ and PM_2.5_ are also found in the Supplementary Material, Table 2.

We calculated lag0, lag1–5 and lag6–10 for cardiovascular and cancer mortality using per 1 µg/m^3^ increment intervals for Model 3 with non-extrapolated 2011 data, Table 3. Associations generally seemed present, and the HRs seemed quite similar across all lags investigated, although for BC, it seemed highest for lag1–5; for example, the HR and 95% CI for BC exposure and cardiovascular mortality at lag1–5 was 2.93 [1.63–5.27] and for lag6–10, 2.35 [1.28 – 4.32], compared to 2.52 [1.41–4.51] for lag0. HRs for NO_x_ remained stable in the same data. In terms of BC exposure and cancer mortality, minor differences between lags were seen, but also here, the HR in the lag1–5 analyses was the highest estimate, with HRs and 95% CIs being 1.44 [0.89–2.32] for lag0, 1.52 [0.94–2.46] for lag1–5 and 1.35 [0.81–2.26] for lag6–10. No major differences between lags were seen for other pollutants.

To evaluate the potential impact of unmeasured confounding, we calculated E-values for the observed associations (Supplementary Material, Table 4). For example, for BC model 3, where the HR with 95% CI was 1.15 [1.06–1.26] per IQR increase, the E-value for the point estimate was 1.57 and 1.30 for the lower confidence limit. This means that an unmeasured confounder would need to be associated with both the exposure and the outcome by an HR of at least 1.31, above and beyond the measured covariates, to fully explain away the observed association. The results suggest that the observed associations are moderately robust to unmeasured confounding, and it is unlikely that such confounding alone would fully account for our findings. As a sensitivity analysis, we restricted mortality follow-up to 2011 for model 3, corresponding to the period for which air pollution exposure estimates were available. The effect estimates were similar to those in the main analysis, although confidence intervals were wider due to the shorter follow-up period and reduced number of events (data not shown). These findings indicate that our main conclusions were robust to this restriction.

## Discussion

This study used a large, robust and well-studied cohort linked with high-quality air pollution data to examine associations between BC exposure and cardiovascular and cancer mortality. After adjustment for relevant confounders, BC exposure was found to be associated with cardiovascular mortality. A tendency toward an association was also observed between cardiovascular mortality and NO_x_ and PM_2.5_. As BC is a component of PM_2.5_, these findings may suggest that BC, *per se* or as a marker for correlated air toxins, represents a particularly strong cardiotoxin, potentially contributing to cardiac mortality previously attributed to PM_2.5_ in other studies. Further research is needed to elucidate these findings. We performed a multi-pollutant analysis to attempt to disentangle BC from PM_2.5_, but the results indicated collinearity issues and therefore not presented.

Cardiovascular toxicity from BC exposure has been suggested in several studies, many of which report clear associations with cardiovascular mortality. Brunekreef et al. (2021) found an HR and 95% CI of 1.085 [1.055–1.116] for cardiovascular mortality per 0.5 × 10^−5^/m related to long-term BC exposure, and an HR and 95% CI of 1.078 [1.033–1.125] for ischaemic heart disease in pooled cohorts [18]. In their administrative cohort, they found HRs and 95% CIs of 1.022 [1.004–1.040] and 1.031 [1.007–1.056] respectively [18]. Vienneau et al. (2023), using the same metric, found an HR and 95% CI of 1.036 [1.022–1.051]; when this was adjusted for PM_2.5_, the HR was 1.034 [1.019–1.049] [22]. Further, Staffogia et al. (2022) found an HR and 95% CI of 1.022 [1.004−1.040] per 0.5 × 10^−5^/m [21]. Weichenthal et al. (2024) found an association, with an HR and 95% CI of 1.015 [1.004−1.027] for cardiovascular mortality per 0.5 µg/m^3^ of BC exposure [23]. Although exposure measures vary collectively, these findings indicate a consistent positive association between BC exposure and cardiovascular mortality, in line with the results of this analysis.

Two studies specifically found associations between BC exposure and ischaemic heart disease mortality, a subset of cardiovascular mortality and a component of the broader cardiovascular outcomes assessed in this cohort. In their pooled cohort, Brunekreef et al. (2021) found an HR and 95% CI of 1.078 [1.033−1.125] for ischaemic heart disease mortality per 0.5 × 10^−5^/m of BC, and in their administrative cohort, an HR and 95% CI of 1.031 [1.007−1.056] [18]. A meta-analysis by Zhu et al. (2023) found an association between BC and ischaemic heart disease mortality, with an HR (RR) and 95% CI of 1.149 [1.024−1.291] [1]. However, interestingly, this analysis did not find a similar positive association for all cardiovascular mortality. Multiple other studies found null associations between cardiovascular mortality [18,19,23,27–29].

We observed tendencies for an association between exposure and cancer mortality, especially with BC. Cancer mortality was included as an outcome given existing evidence of positive associations. Li et al. (2024) examined long-term BC exposure in relation to a range of gastrointestinal cancer outcomes by IQR (0.46 µg/m^3^), finding an overall RR and 95% CI of 1.27 [1.22−1.33], which remained after PM_2.5_ adjustment [26]. They also found evidence for a positive association between BC exposure and oesophageal, stomach, colorectal, hepatic and pancreatic cancers, diagnoses which were captured under our outcome definitions. Also captured under this definition was lung cancer, which has been associated with BC in a 2025 meta-analysis with a pooled HR and 95% CI of 1.10 [1.04–1.17] per 1 µg/m^3^ increase [46]. The findings of this study are based on a low-pollution environment, lower than some other study settings, which may have impacted the statistical power of our findings on cancer mortality. It could be argued that our findings fit with the above literature in that higher levels of exposure produce clearer statistical associations.

One of the major suggested mechanisms for the health impact of BC revolves around immune reactions to inhaled particles. Inflammation, oxidative and nitrosative stress and the subsequent immune cascades triggered by these processes have been implicated across several adverse health outcomes following BC exposure [11,12]. Once BC is inhaled, it can translocate along anatomical structures, disseminate through liquid circulations (such as blood), cross vascular structures (such as the blood–brain barrier, vessel walls and placental tissue) and deposit into tissues (such as brain, lung parenchyma and placenta) [4,13–16]. Local inflammatory responses can then initiate inflammatory cascades leading to structural, physiological and functional alterations, such as through pathological enzyme and cell activation, mitochondrial and cell dysfunction, protein, lipid and DNA damage, leading to impaired repair mechanisms, loss of growth control and altered gene expression, with potential for major histological changes like tissue fibrosis and hyperplasia [4,5,12,13,17,47,48]. Collectively, these biochemical, cellular and histological changes can result in pathological health outcomes, such as those listed above.

Importantly, in relation to the findings of this study, evidence suggests that BC exposure may be linked to major cardiovascular macroscopic changes. Fibrosis, tissue remodelling and hyperplasia are key steps in the development of atherosclerotic plaques, a causal factor in certain forms of ischaemic heart disease leading to cardiovascular mortality, and have been implicated as outcomes of BC exposure alongside inflammatory cascades and platelet activation [4,5,17]. Alteration of endothelial molecules and upregulation of immune responses, particularly in those with existing cardiovascular impairment, may lead to cardiovascular dysfunction in response to BC exposure [4,17]. Some reviews suggest that cardiovascular impact is the most strongly associated adverse health effect that comes from BC exposure, partly due to this increased susceptibility to adverse outcomes in those already suffering cardiovascular impairment or dysfunction [4]. These processes may contribute to the risk of fatal cardiac events. Similarly, altered gene expression, cell dysfunction and loss of growth control, all proposed as effects of BC exposure [4,5,17], are pathological factors implicated in the process of cancer development. Specific effects on DNA function and structure, including breaks, damaged microstructures and damaged repair mechanisms, have been observed in the presence of BC, and these coupled with the presence of metal ions on BC particles give a strong evidence base of an association between BC and cancer [17]. In the present study, we saw less clear associations with cancer mortality than with cardiovascular mortality, both in terms of size of association and the precision of the estimates. The difference in precision of the associations observed for cardiovascular disease versus cancer in this cohort may be partly explained by lower statistical power due to fewer cancer cases. Additionally, the potentially longer latency periods relevant for cancer could also influence the strength of the observed associations, if the critical timing of exposure differs substantially between these outcomes. In the present study, the follow-up period was too short to allow investigation of lag periods longer than 10 years.

BC, as a result of incomplete combustion, has unique physical, chemical and toxicological properties. The surface and structure of BC compounds can be porous and relatively large, meaning that other chemicals and toxins can be attached and absorbed easily [1]. The combustion that generates BC also produces co-pollutants, such as metals, polycyclic aromatic hydrocarbons (PAHs) and other air pollutants, which may accompany BC from emission to absorption, deposition and ultimately to human biochemical and physiological response [1,17,49–52]. This capacity to carry additional toxic substances may enhance the overall toxicity of BC and contribute to its specific health risks as an air pollutant [1].

Outside of the direct health effects, BC can have an impact on the environment and climate, but not primarily as a greenhouse gas; instead, its effects occur through other mechanisms. BC has the ability to impact cloud formation, influencing the climate and water cycle [3]. Further, and especially relevant in colder and/or elevated regions, is BCs ability to deposit onto snow and increase solar radiation absorption [3]. When this occurs, snow, ice and glacier melt may increase, as well as absorbed heat radiation, contributing to increased climate change. It should also be noted that BC is almost always co-emitted with organic carbon (OC) in the incomplete combustion process. OC has a cooling or masking effect on climate change due to its lighter colour. The climate impact of BC depends on its emission source OC/BC ratio [3]. These properties and effects on air pollution, climate and health add importance to the role of BC mitigation.

### Methodological considerations

The strengths of this study include the use of a well-characterised cohort with detailed information on possible confounders and well-defined outcomes. We used an air pollution model based on a comprehensive emission database, and a dispersion model that accounted for meteorological, spatial and temporal variation in exposures. Individual exposure assessment was conducted at each participant’s residential address.

This study has several limitations that should be considered when interpreting the results. First, there is potential for exposure misclassification, as air pollution levels were estimated at participants’ residential addresses and may not fully capture individual-level exposure. This is particularly relevant given the lack of time–activity data, and participants may have spent substantial time away from home (e.g. at workplaces or other locations with different pollution levels). This is standard practice in air pollution epidemiology, and any misclassification can be assumed to be non-differential. Assessing exposure back in time also has some limitations, as the toxic potential by physical properties and chemical compositions of the particles can vary over time. The emission database also became more precise over the years and there are larger uncertainties in the early years. BC was not monitored during the study period and this impacts the possibility to validate the model. Ideally, the BC levels would have been validated for the entire period of the study to provide more clear correlation between modelled levels and measured levels. The emission factors for important sources of BC, such as emissions from vehicles, are however based on well-established methods from EU projects such as TRANSPHORM which aimed at improved emission inventories, measurements of PM and its constituents in European cities [53].

Second, although we adjusted for a wide range of potential confounders, residual confounding remains a possibility. Most major covariates were recorded at baseline and several, such as smoking, physical activity and alcohol consumption, were self-reported, which can introduce reporting bias and measurement error. Changes in these behaviours over time were thus not captured in the analysis. There was no possibility to adjust for traffic noise exposure which can be correlated with NO_x_, which is often seen as a marker for traffic-related air pollution. Similarly, there was no possibility to adjust for ultrafine particles. To assess the robustness of these findings in the light of potential unmeasured confounding, we calculated E-values (see Methods and Discussion) which provide an estimate of the strength of association that an unmeasured confounder would need to have with both the exposure and the outcome to fully explain away the observed associations. The E-values found in this analysis suggest that the observed association, unmeasured confounding beyond the included covariates, should not easily negate the results. This thereby increases confidence in the causal nature of the link between locally emitted BC and cardiovascular mortality. Additionally, the use of E-values increases the generalisability of our results to other environments, including those with differing levels of BC exposure and different ethnic backgrounds. The inclusion of ethnicity (country of birth) in our modelling increases generalisability to environments with different ethnic backgrounds.

Extending our findings to other populations should be done with caution however. Effect estimates may differ depending on factors, such as genetic susceptibility, pollution composition, sociodemographic characteristics and healthcare context. Results may furthermore differ in regions with markedly different environmental or population characteristics. While we have used robust data and statistical methods, adjusted for known confounders and assessed potential residual confounding using E-values, our results are based on exposure levels observed in this cohort. Therefore, we cannot make direct inferences for substantially higher exposure levels than those studied. Assuming a linear exposure–response relationship, as is often observed in air pollution epidemiology, the effects per unit increase in exposure might be similar in other populations. However, the true exposure–response relationship could be non-linear [54], which may influence the magnitude of effects at different exposure levels.

Another limitation is that we only had air pollution exposure estimates of up to 2011, whereas mortality follow-up extended to 2023. As a result, follow-up was limited to 2011 in the main analysis to avoid exposure misclassification, which reduced the statistical power and precision of estimates compared to if there was an air pollution exposure assessment to match the mortality data in follow-up. We used a sensitivity analysis with the last observation carried forward to impute exposures up to 2016, the results changed only marginally, suggesting that the limited exposure window may not have substantially biased the findings. However, greater precision from longer exposure estimates might have enabled detection of clearer associations with cancer mortality.

In this study, we used a short lag period as main results. While it could be argued that a longer lag period may be more appropriate for cancer mortality, we did not find any clearer associations when using other lag periods. This choice is unlikely to have influenced our results.

Our findings on locally emitted BC complement a previous study in this cohort that assessed total BC (including a constant background from long-range transport) [55]. The present study focuses on locally emitted pollutants, which better capture within-city gradients. This methodological difference explains the lower mean BC concentrations reported here and allows us to specifically attribute risk to local emission sources.

## Conclusion

This study examined two leading causes of mortality outcomes – cardiovascular and cancer mortality – using a high-quality cohort and high-resolution air pollution modelling. Our findings, in a relatively low exposure setting, suggest a clear association between BC exposure and cardiovascular mortality and less clear tendencies of associations for cancer mortality. In addition, a focus on locally emitted BC provides and strengthens direct evidence to support urban-level actions and policies for local-source pollution reduction, particularly pollution from traffic, to protect cardiovascular health. These findings add important new data to the growing evidence base showing that long-term exposure to BC increases the risk of cardiovascular mortality especially and strengthens the case for mitigation of BC emissions.

## Supplementary Material

supplementary data file.docx

## Data Availability

Data on air pollution levels are available upon request. Data on individuals cannot be shared due to restrictions in ethical permission.
