# Supplementary material for: Is black carbon associated with cardiovascular and cancer mortality? Evidence from the Malmö Diet and Cancer Cohort
Source: Glob Health Action. 2026 Mar 5;19(1):2636879. doi: 10.1080/16549716.2026.2636879 (PMC12964465; doi:10.1080/16549716.2026.2636879)
Supplement: supplementary data file.docx [file ZGHA_A_2636879_SM4460.docx]

Is Black Carbon a key contributor to cardiovascular and cancer mortality? Evidence from the Malmö Diet and Cancer Cohort

Supplementary Material

Supplement Table 1. Associations between BC, NO_x_ and PM_2.5_ exposure and cardiovascular and cancer mortality, expressed as hazard ratios (HR) with 95% confidence intervals, per 1 µg/m³ increase, based on non-extrapolated data up to 2011, with estimates rounded to 2 decimal places, with two-pollutant model included.

| Cardiovascular mortality | | | |
| --- | --- | --- | --- |
|  | Model 1  n=2103 | Model 2  n=1472 | Model 3  n=1452 |
| BC | 4.31 [2.72 – 6.85] | 2.59 [1.45 - 4.61] | 2.52 [1.41 - 4.51] |
| NO_x_ | 1.01 [1.00 - 1.01] | 1.01 [0.99 - 1.02] | 1.01 [0.99 - 1.02] |
| PM_2.5_ | 1.18 [1.05 - 1.32] | 1.12 [0.97 - 1.31] | 1.12 [0.96 - 1.30] |
| Cancer mortality | | | |
|  | Model 1  n=2586 | Model 2  n=2057 | Model 3  n=2027 |
| BC | 2.11 [1.40 – 3.16] | 1.46 [0.91 - 2.35] | 1.44 [0.89 - 2.32] |
| NO_x_ | 1.00 [0.99 - 1.01] | 1.00 [0.99 - 1.01] | 1.00 [0.99 - 1.01] |
| PM_2.5_ | 1.00 [0.90 - 1.12] | 1.05 [0.92 - 1.19] | 1.04 [0.91 - 1.18] |

“n” refers to the number of complete cases for the specified outcome. BC - Black Carbon. NO_x_ - Nitrogen oxides. PM_2.5_ - fine particulate matter. Results were presented as a 1 µg/m³ increase in each pollutant. Model 1: year of birth and sex; Model 2: additional adjustment for employment status, education, physical activity score, smoking status, BMI, marital status, waist-hip ratio, alcohol consumption, year of enrolment, study year, and country of birth; Model 3: further adjustment for smoking pack-years and vegetable consumption.

Supplement Table 2. Associations between BC, NO_x_ and PM_2.5_ exposure and cardiovascular and cancer mortality, expressed as hazard ratios (HR) with 95% confidence intervals, per 1 µg/m³ increase, based on extrapolated data up to 2016, with estimates rounded to 2 decimal places, with two-pollutant model included.

| Cardiovascular mortality | | | |
| --- | --- | --- | --- |
|  | Model 1  n=2135 | Model 2  n=1496 | Model 3  n=1476 |
| BC | 3.38 [2.14 – 5.33] | 2.64 [1.49 - 4.68] | 2.57 [1.44 - 4.57] |
| NO_x_ | 1.01 [1.00 - 1.01] | 1.01 [0.99 - 1.02] | 1.01 [0.99 - 1.02] |
| PM_2.5_ | 1.08 [0.96 - 1.22] | 1.13 [0.98 - 1.31] | 1.12 [0.97 - 1.31] |
| Cancer mortality | | | |
|  | Model 1  n=2613 | Model 2  n=2079 | Model 3  n=2049 |
| BC | 1.75 [1.17 - 2.62] | 1.48 [0.92 - 2.37] | 1.46 [0.91 - 2.34] |
| NO_x_ | 1.00 [0.99 - 1.01] | 1.00 [0.99 - 1.01] | 1.00 [0.99 - 1.01] |
| PM_2.5_ | 0.94 [0.84 - 1.04] | 1.05 [0.93 - 1.19] | 1.04 [0.92 - 1.18] |

“n” refers to the number of complete cases for the specified outcome. BC - Black Carbon. NO_x_ - Nitrogen oxides. PM_2.5_ - fine particulate matter. Results were presented as a 1 µg/m³ increase in each pollutant. Model 1: year of birth and sex; Model 2: additional adjustment for employment status, education, physical activity score, smoking status, BMI, marital status, waist-hip ratio, alcohol consumption, year of enrolment, study year, and country of birth; Model 3: further adjustment for smoking pack-years and vegetable consumption.

Supplement Table 3. Associations between BC, NO_x_ and PM_2.5_ exposure and cardiovascular and cancer mortality, expressed as hazard ratios (HR) with 95% confidence intervals, per IQR increase, using extrapolated data up to 2016, with estimates rounded to 2 decimal places, with two-pollutant model included.

| Cardiovascular mortality | | | |
| --- | --- | --- | --- |
|  | Model 1  n=2135 | Model 2  n=1496 | Model 3  n=1476 |
| BC | 1.21 [1.13 - 1.30] | 1.16 [1.06 - 1.27] | 1.16 [1.06 - 1.27] |
| NO_x_ | 1.09 [1.02 - 1.17] | 1.09 [0.99 - 1.19] | 1.09 [0.99 - 1.19] |
| PM_2.5_ | 1.05 [0.98 - 1.25] | 1.09 [0.98 - 1.20] | 1.08 [0.98 - 1.20] |
| Cancer mortality | | | |
|  | Model 1  n=2613 | Model 2  n=2079 | Model 3  n=2049 |
| BC | 1.09 [1.03 - 1.16] | 1.06 [0.99 - 1.14] | 1.06 [0.98 - 1.14] |
| NO_x_ | 1.01 [0.95 - 1.08] | 1.03 [0.95 - 1.11] | 1.02 [0.95 - 1.10] |
| PM_2.5_ | 0.96 [0.89 - 1.03] | 1.03 [0.95 - 1.12] | 1.03 [0.94 - 1.12] |

“n” refers to the number of complete cases for the specified outcome. BC - Black Carbon. NO_x_ - Nitrogen oxides. PM_2.5_ - fine particulate matter. Results were presented as IQR increase for each pollutant, which is 0.12 µg/m³ for BC, 6.05 µg/m³ for NO_x_ and 0.56 µg/m³ for PM_2.5_. Model 1: year of birth and sex; Model 2: additional adjustment for employment status, education, physical activity score, smoking status, BMI, marital status, waist-hip ratio, alcohol consumption, year of enrolment, study year, and country of birth; Model 3: further adjustment for smoking pack-years and vegetable consumption.

Supplement Table 4. E-Values for significant associations between BC, NO_x_ and PM_2.5_ exposure lag0 and cardiovascular and cancer mortality, expressed as hazard ratios (HR) with 95% confidence intervals, per IQR increase, based on non-extrapolated data up to 2011, with estimates rounded to 2 decimal places.

| Cardiovascular mortality | | | |
| --- | --- | --- | --- |
|  | Model 1  n=2103 | Model 2  n=1765 | Model 3  n=1745 |
| BC | 1.26 [1.17 - 1.36]  E-value point estimate: 1.83 E-value lower CI-limit: 1.62 | 1.15 [1.06 - 1.25]  EV point: 1.57  EV lower: 1.31 | 1.15 [1.06 - 1.25]  EV point: 1.57  EV lower: 1.31 |
| NO_x_ | 1.17 [1.09 - 1.26] EV point: 1.62  EV lower: 1.40 | Not applicable | Not applicable |
| PM_2.5_ | 1.14 [1.05 - 1.24]  EV point: 1.54 EV lower: 1.28 | Not applicable | Not applicable |
| Cancer mortality | | | |
|  | Model 1  n=2587 | Model 2  n=2314 | Model 3  n=2284 |
| BC | 1.09 [1.03 - 1.17]  EV point: 1.40  EV lower: 1.21 | Not applicable | Not applicable |
| NO_x_ | Not applicable | Not applicable | Not applicable |
| PM_2.5_ | Not applicable | Not applicable | Not applicable |

“n” refers to the number of complete cases for the specified outcome. BC - Black Carbon. NOx - Nitrogen oxides. PM_2.5_ - fine particulate matter. Results were presented as IQR increase for each pollutant, which is 0.12 µg/m³ for BC, 6.05 µg/m³ for NO_x_ and 0.56 µg/m³ for PM_2.5_. Model 1: year of birth and sex; Model 2: additional adjustment for employment status, education, physical activity score, smoking status, BMI, marital status, waist-hip ratio, alcohol consumption, year of enrolment, study year, and country of birth; Model 3: further adjustment for smoking pack-years and vegetable consumption.

Supplementary Table 5. Missing data for key variables.

| Variables | All cohort | Cardiovascular mortality | | Cancer mortality | |
| --- | --- | --- | --- | --- | --- |
|  |  | No | Yes | No | Yes |
|  | n=30440 | n=28305 | n=2135 | n=27826 | n=2614 |
| Continuous variables – number (%) | | | | | |
| Physical activity  score | 2085 (6.85) | 1850 (6.54) | 235 (11.01) | 1899 (6.82) | 186 (7.12) |
| BMI | 52 (0.17) | 45 (0.16) | 7 (0.33) | 49 (0.18) | 3 (0.11) |
| Waist/hip ratio | 68 (0.22) | 60 (0.21) | 8 (0.37) | 64 (0.23) | 4 (0.15) |
| Alcohol consumption (g/day) | 2210 (7.26) | 1981 (7.00) | 229 (10.73) | 2022 (7.27) | 188 (7.19) |
| Smoking pack years | 2235 (7.34) | 2003 (7.08) | 232 (10.87) | 2041 (7.33) | 194 (7.42) |
| Vegetable consumption | 2210 (7.26) | 1981 (7.00) | 229 (10.73) | 2022 (7.27) | 188 (7.19) |
| Categorical variables – number (%) | | | | | |
| Sex | 0 (0.0) | 0 (0.0) | 0 (0.0) | 0 (0.0) | 0 (0.0) |
| Education level | 1946 (6.4) | 1727 (6.1) | 219 (10.3) | 1782 (6.4) | 164 (6.3) |
| Cohabiting  w. partner | 1890 (6.2) | 1679 (5.9) | 211 (9.9) | 1726 (6.2) | 164 (6.3) |
| Current employment | 2485 (8.2) | 2197 (7.8) | 288 (13.5) | 2267 (8.1) | 218 (8.3) |
| Smoking | 1881 (6.2) | 1667 (5.9) | 214 (10.0) | 1718 (6.2) | 163 (6.2) |
| Birth country | 0 (0.0) | 0 (0.0) | 0 (0.0) | 0 (0.0) | 0 (0.0) |
| Year of enrolment |  | 3789 (13.4) | 347 (16.3) | 3844 (13.8) | 292 (11.2) |

Percentages are listed column-wise for the specified categorical variable per outcome. “n” refers to the number of complete cases for the specified outcome. ^a^ The word “housewife” has been kept as it was asked in the original survey, though it is outdated terminology now. BMI - Body Mass Index, g - grams

Supplement Figure 1. Pearson coefficient and collinearity between BC, PM_2.5_, particulate matter (PM_10_) and NO_x_ pollutants from this dataset.


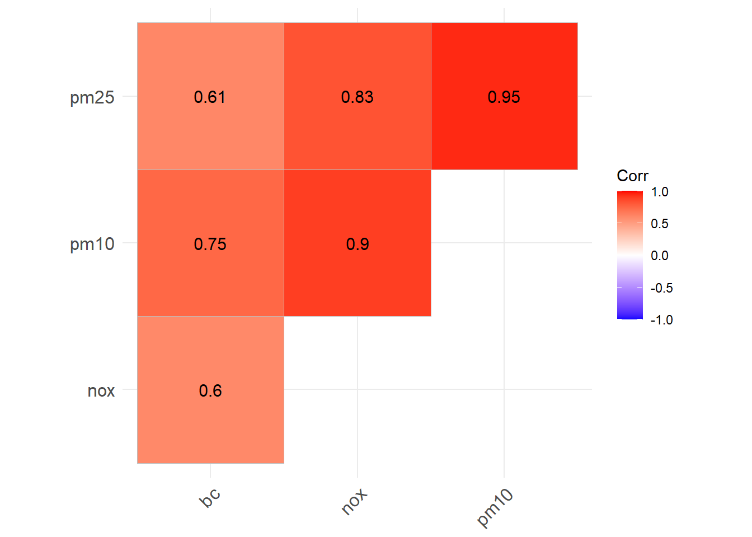

Note: “bc” refers to black carbon, “pm25” refers to PM_2.5_, “pm10” refers to PM_10_, “nox” refers to NO_x_, “corr” refers to the degree of correlation.

Supplement Figure 2. Missing data among covariates.
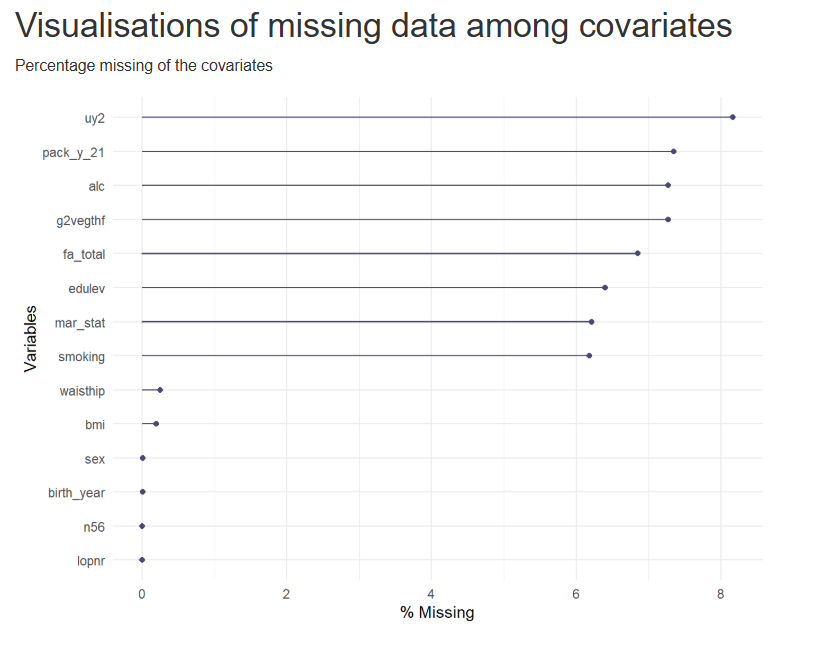


Note: uy2 (birth country), pack_y_21 (Smoking pack years), Fa_total (Physical activity score), g2vegthf (Vegetable consumption), alc (Alcohol consumption (g/day)), edulev (Education level), mar_stat (Cohabiting w. partner), waisthip (Waist/hip ratio), bmi (BMI), n56 (current employment).
